# Clinical study of headache in relation to sinusitis and its management

**Published:** 2013-12-25

**Authors:** A Kaur, A Singh

**Affiliations:** * Department of Physiology, M.M Institute of Medical Sciences and Research, Mullana (Ambala); ** Department of E.N.T., M.M Institute of Medical Sciences and Research, Mullana (Ambala)

**Keywords:** Headache, Sinusitis, FES

## Abstract

Abstract

Aim: To study relation of headache with sinusitis and its management. Methodology: Patients clinically presenting with headache were selected. Only patients with headache due to rhinogenic causes were subjected to X-ray paranasal sinuses and diagnostic nasal endoscopy and followed-up to evaluate management. Results: Majority of the patients were of age group 21-30 years and it is more predominant in males. Majority of the patients of headache were having DNS (28.9%), acute sinusitis (28.9%), osteomeatal complex disease (24.63%) and few number of patients had nasal polyp (8.69%), allergic rhinitis (5.79%) and rarely patients had atrophic rhinitis (2.89%). Headache was localized in forehead (43.4%), more than one site (34.7%) in majority of cases and few number of patients had headache at glabella (13.04%) and top of head (8.69%). Majority of the patients who underwent antral washout were not relieved, so they underwent functional endoscopic sinus surgery, which gave dramatic results in improving symptoms of patients including headache. Conclusions: Headache is nearly a universal human experience. The lifetime incidence of headache is estimated to be at least 90%. To know whether the headache is sinogenic or not; firstly the patient is clinically assessed, then radiological investigations (X-ray PNS) are done. Role of FESS is huge and ultimately it is it that is the cure for the headache due to rhinogenic causes. Abbreviations: DNE - Diagnostic nasal endoscopy, FESS - Functional endoscopic sinus surgery, PNS - Paranasal sinuses

## Introduction

Headache is nearly a universal human experience. The lifetime incidence of headache is estimated to be at least 90%. Moskowitz has described headache as the symptom produced by the nervous system when it perceives threat and as such is considered part of the protective physiology of the nervous system. When the cause of headache is a definable underlying pathologic process, the headache is diagnosed as a secondary headache. Causes include metabolic, infectious, inflammatory, traumatic, neoplastic, immunologic, endocrinologic and vascular entities. When no clear pathologic condition can be identified, headache is considered to be a manifestation of a primary headache syndrome. The common primary headache disorders as defined by the International Headache Society, are migraine, probable migraine, tension type and cluster headache [**1**].Our aim is to study the relation of headache in sinusitis. The term "sinusitis" refers to a group of disorders characterized by inflammation of the mucosa of the paranasal sinuses. Because the inflammation nearly always also involves the nose, it is now generally accepted that "rhinosinusitis" is the preferred term to describe the inflammation of the nose and paranasal sinuses [**2**]. Patients with chronic headache pain often present to a variety of specialists, including their primary care physician, neurologist, dentist, otolaryngologist and even psychiatrist. They present to otolaryngologist because they or their physician believe the headache to be related to underlying sinus pathology. The primary focus of the otolaryngologist is to exclude this possibility. The diagnosis of headache secondary to acute-sinusitis can be relatively straightforward. Diagnosing headache related to chronic sinus disease can be much more difficult depending on patients’ presentation [**3**]. Endoscopic techniques are now well established. In combination with modern imaging techniques particularly CT; these techniques provide diagnostic possibilities unimagined a few decades ago [**4**].

## Materials and methods 

The present study "clinical study of headache in relation to sinusitis and its management" was conducted in the Department of Otorhinolaryngology in M.M Medical College and Hospital, Mullana, Ambala from October 2009 to July 2011.

 Source of Data

 Patients for the study were collected from the Department of Otorhinolaryngology, M.M Medical College and Hospital, Mullana, Ambala.

Sample Size

The study included 100 patients and the cases were diagnosed based upon the clinical examination and investigation.

 Inclusion Criteria

 Patients presenting with clinical features of sinusitis of all age groups and sexes.

 Exclusion Criteria

 All patients presenting with clinical features other than sinusitis.

 These patients were evaluated as follows:

 • Selected patients were subjected to a complete examination according to a defined proforma.

 • Detailed history with thorough clinical examination was done.

 • Patients were asked about history of headache.

 1. Mode of onset

 2. Duration of complaint

 3. Continuous or intermittent

 4. Progressive or not

 5. Site of pain and radiation

 6. Type of pain

 7. Associated symptoms.

 8. Aggravating and relieving factors

 9. Duration of each attack.

 10. Frequency of attack

 11. Time of onset of attack.

 12. Treatment taken for the same.

 Then if the headache was suspected of rhinogenic or sinogenic origin, the patients underwent detailed otorhinolaryngological examination.

 • Routine blood investigations like Hb, TC, DC, ESR, BT, CT urine for albumin, sugar and microscopy.

 • Radiological investigations i.e., X-ray paranasal sinuses (waters view) was advised in all patients of headache due to rhinogenic or sinogenic origin.

 • DNE was advised to the same group of patients.

 • Acute infections were first treated with medicines.

 • Patients having haziness of maxillary sinuses on PNS X-ray were advised antral wash.

 • Patients who were having haziness of frontal sinuses and the patients who were not relieved of headache after antral washout were advised FESS.

 Observations

 A total of 100 patients with headache were studied for a period of about 2 years i.e., form October 2009 to July 2011, of which only 69 patients had headache due to rhinogenic causes.

**Table 1:** Age distribution (Rhinogenic Causes) (n=69)

	Age group (years) 5 – 10	11 - 20	21 – 30	31 – 40	41 – 50	51 – 60
DNS	-	6	14	-	-	-
Acute sinusitis	-	8	12	-	-	-
Osteomeatal complex disease	-	7	10	-	-	-
Polyp	1	-	5	-	-	-
Allergic rhinitis	-	4	-	-	-	-
Atrophic rhinitis	-	-	-	2	-	-

 The highest age incidence is present in the age group of 21-30 years (59.42%), followed by 11-20 years (36.23%).

**Fig. 1:**
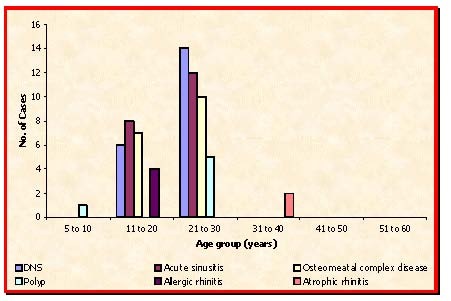
Age distribution (Rhinogenic Causes)

**Fig. 2:**
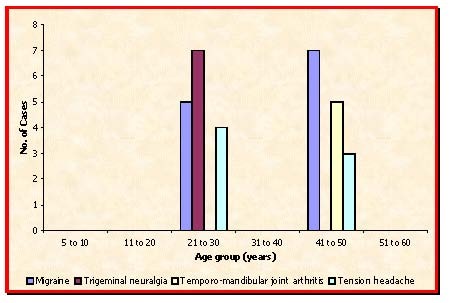
Age Distribution (others)

**Table 2:** Age Distribution (others) (n=31)

	Age group (years) 5 – 10	11 - 20	21 – 30	31 – 40	41 – 50	51 – 60
Migraine	-	-	5	-	7	-
Trigeminal neuralgia	-	-	7	-	-	-
Temporo-mandibular joint arthritis	-	-	-	-	5	-
Tension headache	-	-	4	-	3	-

On headache due to other causes, patients of age group 21-30 years are more prone (51.61%). 

**Table 3:** Sex distribution (Rhinogenic causes) (n=69)

	Male	Female
DNS	14	6
Acute sinusitis	8	12
Osteomeatal complex disease	10	7
Polyp	1	5
Allergic rhinitis	4	0
Atrophic rhinitis	-	2

 53.62% of the patients with headache due to rhinogenic causes were males and 46.37% were females

**Fig. 3:**
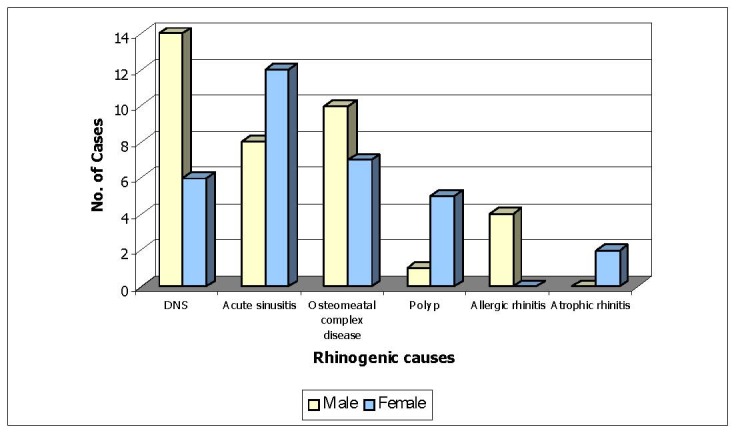
Sex distribution (Rhinogenic causes)

**Table 4:** Sex Distribution (Other causes) (n=31)

	Male	Female
Migraine	2	10
Trigeminal neuralgia	-	7
Temporo-mandibular joint arthritis	-	5
Tension headache	-	7

 93.54% of the patients with headache due to other causes were females and 6.45% were males.

**Fig. 4:**
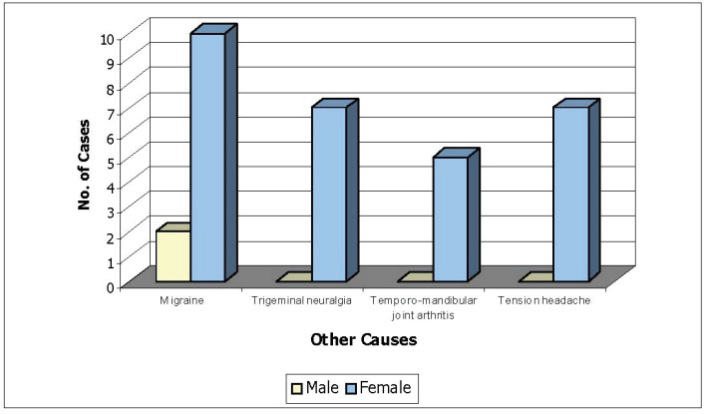
Sex Distribution (Other causes)

**Table 5:** Etiology of Headache with Respect to Clinical Findings

	No. of cases	Percentage of Rhinogenic causes
DNS	20	28.90
Acute sinusitis	20	28.90
Osteomeatal complex disease	17	24.63
Polyp	6	8.69
Allergic rhinitis	4	5.79
Atrophic rhinitis	2	2.89

 28.9% of the patients with headache had DNS and 28.9% of the patients had acute sinusitis, 24.63% of the patients had osteomeatal complex disease and 8.69% of patients had polyp, 5.79% of patients had allergic rhinitis and 2.89% of patients had atrophic rhinitis.

**Fig. 5:**
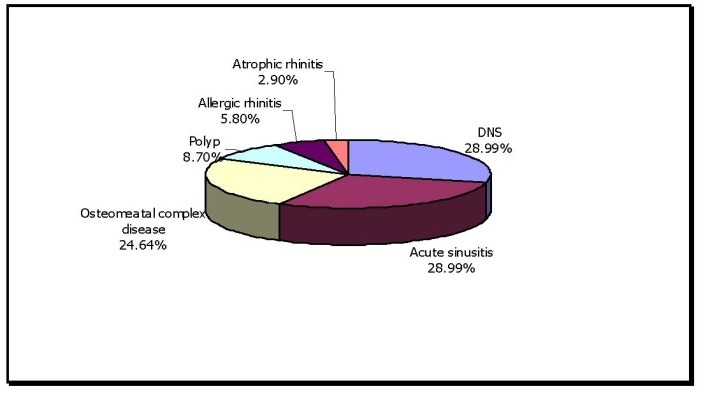
Etiology of headache with respect to clinical findings

**Table 6:** Localization of headache

Localization	No. of cases	Percentage of Rhinogenic causes
Forehead	30	43.4
More than one site	24	34.7
Glabella	9	13.04
Top of head	6	8.69

 Patients with headache in forehead were maximum i.e., 43.4% followed by headache at more than one site 34.7% and then glabella 13.04% and top of head 8.69%.

**Fig. 6:**
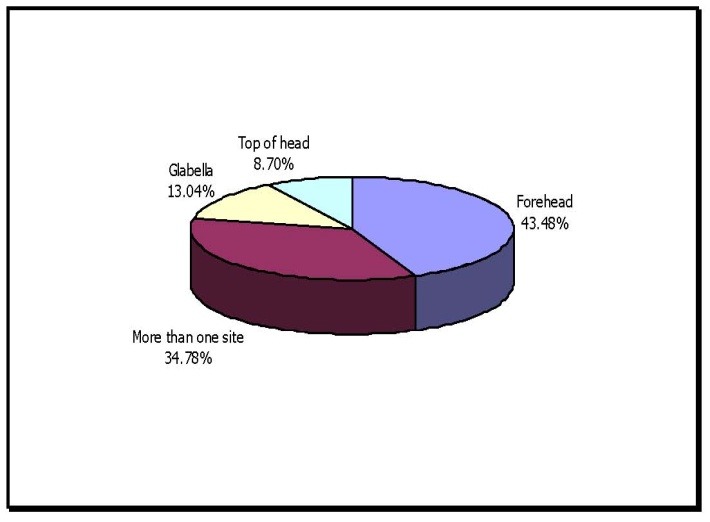
Localization of headache

**Table 7:** Patients who underwent antral washout

	No. of patients	Relieved (n=20)	Not relieved (n=37)
DNS	20	8	12
Acute sinusitis (under antibiotic cover)	20	5	15
OMD	17	7	10

35.08% of patients who underwent antral washout for headache and facial pain were relieved, whereas 64.92% were not relieved.

**Fig. 7:**
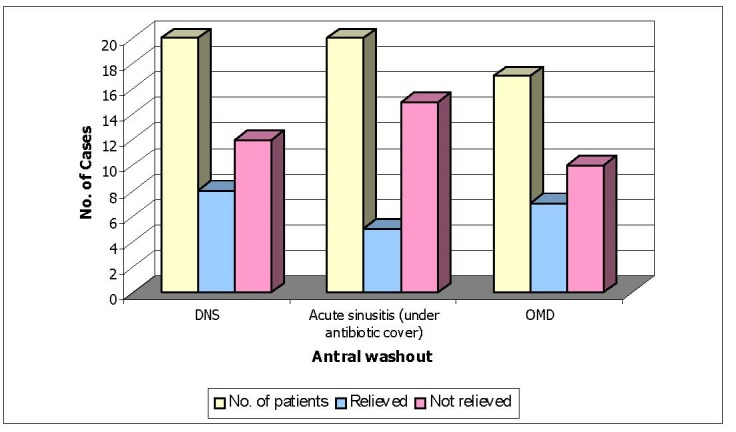
Patients who underwent antral washout

**Table 8:** Patients who underwent DNE (n=69)

	No. of cases	Percentage
Mucosal contact points present	30	43.47
Mucosal contact points absent	39	56.52

 43.47% of the patients who underwent DNE for headache had mucosal contact points as the main pathology.

**Table 9:** Patients who underwent FESS due to mucosal contact point (n=25)

	No. of cases	Percentage
Total relief from headache	20	80.00
Significant relief	5	20.00

 Out of 30 patients diagnosed as having mucosal contact points, 25 underwent FESS. 80% of the patients who underwent FESS for headache due to mucosal contact points were relieved totally from headache and 20% had significant relief.

**Table 10:** Patients who underwent FESS due to causes other than Contact Points (n=30)

	No. of cases	Percentage
Completely free of pain	9	30.00
Significant symptom improvement	11	36.6
No benefit from surgery	10	33.3

 Out of 39 patients of headache due to causes other than contact point, 30 underwent FESS. Patients who underwent FESS for causes other than mucosal contact points showed 67% improvement in headache and facial pain.

**Fig. 8:**
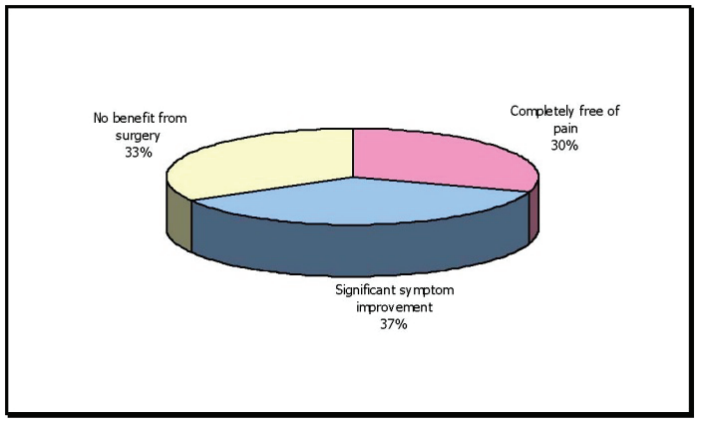
Patients who underwent FESS due to causes other than Contact Points

## Discussion

 According to our study the majority of the cases of headache due to rhinogenic causes were males (54%) in the age group of 11-30 years. The same findings are present in the literature in the study conducted by Pramod Kumar et al 2000 showing majority of patients of headache belonging to age group 10-30 years and 53% were males [**5**]. Similarly in a different study by Wenig et al. and Lebovics et al. demonstrated a male predominance of headache due to acute frontal sinusitis in both adults and adolescents [**6**]. 

 While the majority of our patients with headache due to rhinogenic causes were having either D.N.S, Acute sinusitis or Osteomeatal complex disease, we also encountered patients having nasal polyps (six patients) allergic rhinitis (four patients) presenting with headache. Similarly, in the study by Junior DeFrietas, the patients of polyps along with nasal obstruction also had facial discomfort and headache [**7**] and according to a study conducted by Wolf, 20% of allergy patients presented with headache [**8**]. In our study, out of 69 patients of headache due to rhinogenic causes, 30 patients had headache at forehead i.e., 43.4%, 24 patients had headache at more than one site i.e., 34.7%, 9 patients had headache at glabella i.e., 13.04% and 6 patients had headache at top of head i.e., 8.69%. In a study conducted by Pramod Kumar et al (2000), localization of headache to forehead was 43% while headache at more than one site was 19%, pain at glabella 12% and headache at top of head was 9% [**5**]. 

 Thus, it concludes that headache is localized at forehead in majority of cases.

 We also did antarl washouts in our patients of headache but only 35% of the patients were relieved. 

 We also did D.N.E on 69 patients out of which 30 patients had mucosal contact points present (43%). Patients due to mucosal contact points were advised to undergo functional endoscopic sinus surgery. Out of 30 patients, 25 patients underwent surgery. Post-operatively, 20 patients (80%) had total relief from headache, 5 patients (20%) had significant relief. Various other studies are present in literature, which show the same results. In a study conducted by Behin F, Behin B, 23 patients underwent surgical intervention to relieve the contact points.83% of patients no longer complained of headache. 8% had significant relief [**9**]. 

 In a study conducted by Parsons DS, Batra PS on 34 patients who underwent surgery for contact points reported a reduction in intensity in 91% of patients and reduction in frequency of headache in 85% of patients postoperatively [**10**]. Thus, from our study and the above mentioned studies, it is clear that majority of the patients who underwent FESS for mucosal contact points are totally relieved of the symptoms. The remaining 39 patients who had pathologies other than mucosal contact points i.e., DNS, osteomeatal complex disease were also advised FESS to get rid of headache.

 Out of which 30 patients underwent surgery.

 Post-operatively, 9 patients were completely free of pain – 30%; 11 patients had significant symptom improvement – 36.6% and 10 patients had no benefit from surgery – 33.3%.

Thus, it showed 67% improvement and correlates with studies mentioned below: In a study conducted by Welge-Leussen A et al, 10 years follow up of the patients who had undergone FESS was done. Out of 20 patients, six patients remained completely free of pain (30%), seven had significant improvement (35%) and seven (35%) received no benefit from surgery (65% improvement) [**11**]. 

 In a study conducted in Department of Otolaryngology, Vajira Hospital, Bankok, 16 patients were operated on by FESS. Their principal complaint was facial pain or headache. Ten patients had no headache postoperatively (62.5%) and six patients (37.5%) had a reduction in severity [**12**]. 

 Thus, from the present study and the above mentioned studies, an improvement in headache in 63 – 67% of patients operated should be expected after the patients undergoes FESS for headache.

## Conclusion

Sinusitis refers to a group of disorders characterized by inflammation of the mucosa of the paranasal sinuses. Now-a-days rhinosinusitis is the preferred term to describe the inflammation of the nose and paranasal sinuses. Headache is nearly a universal human experience. The lifetime incidence of headache is estimated to be at least 90%.

 Before treating the headache it should be known that whether the headache is primary (when no clear pathologic condition can be identified) or secondary (metabolic, infectious, inflammatory, traumatic, neoplastic, immunologic, endocrine, vascular).

 Knowing whether the headache is sinogenic or not, firstly the patient is assessed clinically, then radiological investigations (X-ray PNS) are done. Patients also undergo diagnostic nasal endoscopy. Medical line of treatment with antibiotics, antihistaminics, anti-inflammatory, nasal decongestant will be beneficial only in acute cases of sinusitis without any anatomical variation.

 Most cases of sinusitis presenting with headache are acute cases or acute or chronic sinusitis. 

 Antral lavage can be a relief from headache for some patients.

 The role of FESS is huge when no obvious clinical abnormality is made out and ultimately it is FESS that is the cure for headache due to rhinogenic causes.

 Nowadays, suction irrigation endoscopy should be used for visual control during surgery. Microdebrider should be used for FESS as it provides atraumatic dissection with minimum bleeding, which enables decreased surgical time and faster postoperative healing.

Summary

 1. A total of 100 patients presenting in ENT Department with headache were taken for study.

2. Out of 100 patients, 69 patients had headache due to sinogenic causes.

 3. Headache can occur at any age. But the highest incidence was noted in the age group 21-30 years followed by 11-20 years.

 4. Sex incidence is slightly more in males (53.62%).

 5. In the patients of headache due to sinogenic cause 20 patients (28.9%) had acute sinusitis, 20 patients (28.9%) had DNS, 17 patients (24.63%) had osteomeatal complex disease (OMD), 6 patients (8.69%) had polyps, 4 patients (5.79%) had allergic rhinitis and 2(2.89%) had atrophic rhinitis.

 6. Headache was localized to forehead in 30 patients (43.4%) and more than one site in 24 patients (34.7%), at the glabella in 9 patients (13.04%) and in 6 patients (8.69%) at the top of head.

 7. Out of 69 patients of headache due to sinogenic causes, 57 underwent antral washout, 20 patients were relieved of their headache and facial pain (35.08%) and 37 patients (64.9%) were not relieved.

 8. All the 69 patients underwent diagnostic nasal endoscopy out of which 30 patients (43.47%) were found to have mucosal contact points.

 9. Out of 30 patients of headache due to mucosal contact points, 25 underwent FESS, out of which 20 patients (80%) had total relief from headache and 5 patients (20%) had significant relief.

 10. Out of the remaining 39 patients of headache 30 patients underwent FESS, 9 patients (30%) had complete relief from pain, 11 patients (36.6%) had significant symptom improvement, 10 patients (33.3%) had NO benefit from surgery. It showed that 67% of patients had improvement of headache after undergoing FESS.

 11. Endoscopic management of headache due to sinogenic causes provides a tool to the surgeon by which he can accurately diagnose meticulously and with minimal trauma operate and precisely provide post operative care and follow-up.

 12. The use of microdebrider provides an excellent surgical result with fewer complications and faster healing than traditional techniques in FESS.

Source of support- NIL

 Conflict of interest-NONE DECLARED
